# Are Dyslipidemias a risk factor for Peri-Implant complications? A Systematic review and meta-analysis

**DOI:** 10.4317/jced.62241

**Published:** 2025-04-01

**Authors:** Barbara Gurgel, Roberta Souza, André Vajgel, Maria Fernanda Cabral, Marina Almeida, Renata Almeida

**Affiliations:** 1 Student, PhD, Department of Oral and Maxillofacial Surgery University of Pernambuco – School of Dentistry (UPE/FOP); 2 Student, MSc, Specialist in Oral and Maxillofacial Surgery, Department of Oral and Maxillofacial Surgery University of Pernambuco – School of Dentistry (UPE/FOP); 3 PhD, Specialist in Oral and Maxillofacial Surgery, Professor of University of Pernambuco – School of Dentistry, Arcoverde-PE; 4 Student, University of Pernambuco – School of Dentistry, Arcoverde-PE; 5 Student, University Pernambucana of Health - Medicine; 6 PhD in Oral and Maxillofacial Surgery, Professor of University of Pernambuco, Department of Oral and Maxillofacial Surgery

## Abstract

**Background:**

Dyslipidemia, characterized by high levels of lipids in the blood, can influence bone metabolism, interfere with the osseointegration of dental implants, and favor peri-implant complications. This systematic review aimed to analyze whether elevated serum levels of total cholesterol and its fractions, as well as triglycerides, constitute a risk factor for peri-implant complications.

**Material and Methods:**

This systematic review was conducted by PRISMA and was registered in PROPSPERO under CRD number 42023456517. The search strategy was performed in the Medline (Pubmed), Embase, Scopus, Web of Science, Cochrane Library, and LLACS/BVS databases. Additional searches were conducted in the gray literature, as well as manual searches. Experimental and observational studies of patients undergoing dental implants, in which their lipid levels were analyzed and in which the outcomes of peri-implantitis, implant loss, or bone resorption were evaluated. The assessment of methodological quality was performed using the JBI Instrument, and the assessment of the certainty of the evidence was performed using GRADE.

**Results:**

The database search strategy resulted in 2,714. After removing duplicates and reading titles and abstracts, 48 were read in full. Of these, eight were included in the systematic review, in addition to three articles included from additional searches, totaling 11 studies, and a total of 1704 patients constituted the sample. Nine studies identified a significant association between elevated lipid levels and peri-implant complications. The meta-analysis demonstrated a strong association between peri-implantitis and dyslipidemia (OR 12.36; 95% CI: 7.85–19.46; *P*< 0.00001).

**Conclusions:**

Dyslipidemia was identified as a risk factor for peri-implant complications. This can be explained by the interference that high levels of cholesterol and its fractions can cause in bone metabolism, making it deficient due to an exacerbated inflammatory response. Cholesterol and triglyceride levels must be assessed and controlled preoperatively to reduce peri-implant complications.

** Key words:**Dyslipidemias, Hyperlipidemias, Dental implants, Peri-Implantitis, Systematic Review.

## Introduction

Implant dentistry is frequently recommended for the treatment of patients with partial or complete edentulism (1). Despite the success of implants and their excellent long-term prognosis, the prevalence of peri-implant diseases has increased. The survival rate of dental implants depends primarily on successful osseointegration after their placement (2). Any alteration of this biological process can adversely affect the survival rate.

According to some authors, risk factors for implant failure are related to both the individual’s systemic health, such as diseases like diabetes, hypertension, heart problems, gastric issues, osteoporosis, hypothyroidism, or hyperthyroidism, as well as their lifestyle habits, such as smoking. In addition to these conditions, some recent studies have suggested that there may be a relationship between hyperlipidemia and osseointegration of dental implants (3-6).

Hyperlipidemia is a group of diseases in which the level of plasma lipids measured through peripheral blood collection increases beyond accepTable limits, exceeding the normal range (7). In general, dyslipidemia has traditionally been defined as any alteration in the level of lipoproteins (including cholesterol, triglycerides, and lipids). It is often evidenced by one or more of the following: total cholesterol > 190 mg/dL, LDL cholesterol > 130 mg/dL, HDL cholesterol <40 mg/dL, or triglycerides > 200 mg/dL (8).

According to Sun *et al*. (2023) (9), in a hyperlipidemic environment, the titanium implant itself does not favor osseointegration because, in the presence of oxidative lipids, there is an increase in osteoclast activity and bone resorption, along with the induction of local inflammation, impairing osseointegration.

Determining whether there is a relationship between the effects of hyperlipidemia and hypercholesterolemia on peri-implant health or disease, considering evidence of a possible association between them, has become a clinical question that needs to be answered. Therefore, this systematic review aimed to analyze whether elevated serum levels of total cholesterol and its fractions, as well as triglycerides, constitute a risk factor for peri-implant complications.

## Material and Methods

**Figure 1:**
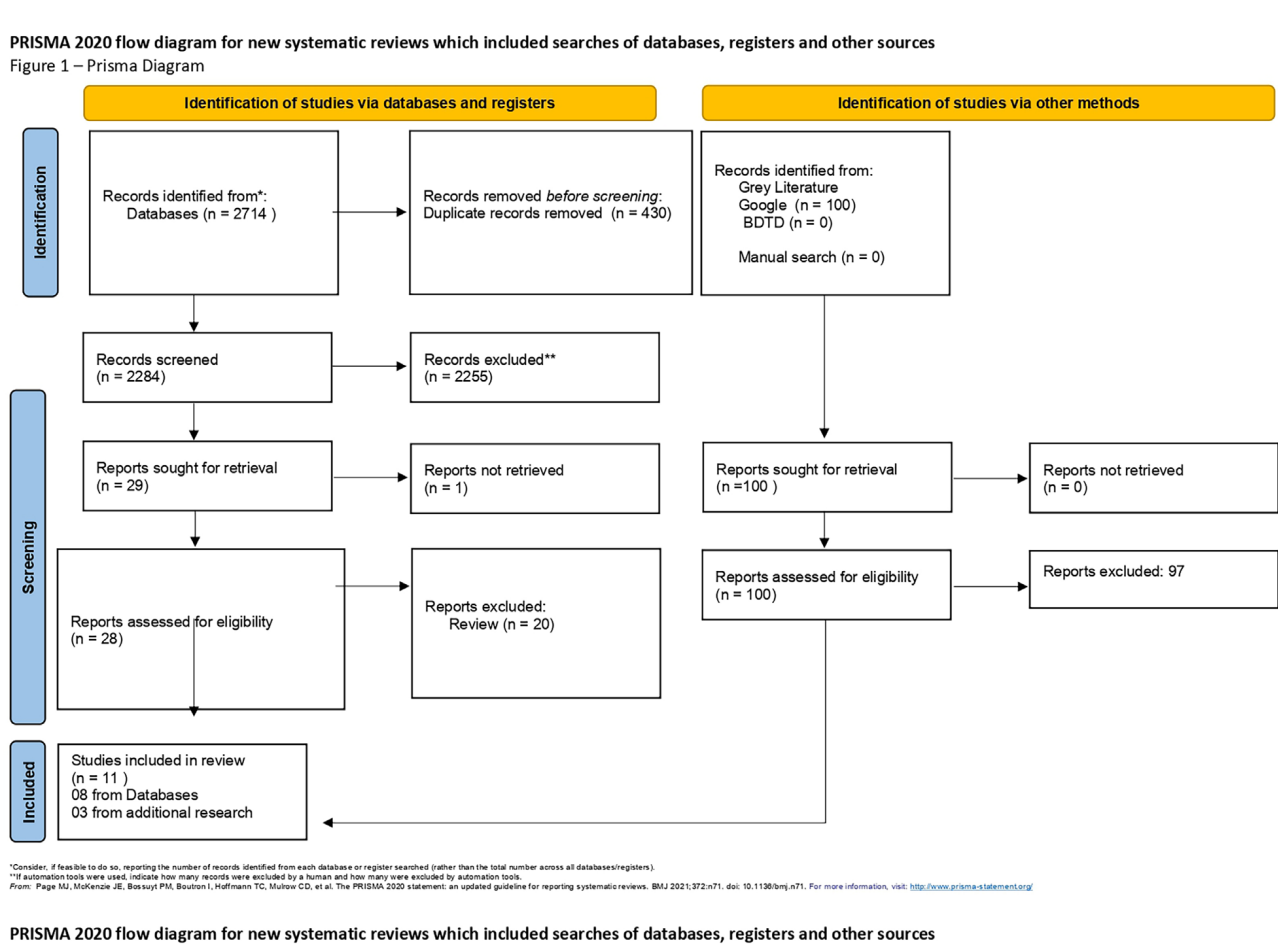
PRISMA Diagram.

This systematic review was conducted following the guidelines of the Cochrane Handbook (10) and PRISMA (11). (Fig. 1 - Prisma Diagram); and has been registered with the International Prospective Register of Systematic Reviews (PROSPERO) and is approved under protocol number CRD42023456517.

-Focused question

Based on the PECO strategy—population (adult patients undergoing dental implant placement); exposure (elevated levels of total cholesterol, LDL, HDL, and triglycerides—dyslipidemia); comparator (patients with normal cholesterol and triglyceride levels); and outcome (peri-implant complications such as peri-implantitis, implant loss, and bone resorption)—the following focused question was proposed: “Is dyslipidemia a risk factor for peri-implant complications?”

-Study selection

Two researchers (RA and BR) independently and blindly conducted the article selection process. The selection occurred in two stages: first, reading titles and abstracts to identify articles important for addressing the guiding question based on eligibility criteria; second, a full study reading to select articles for systematic review, from which data will be extracted. Consensus meetings were held at each selection stage to resolve conflicts between reviewers. The article selection followed a two-step process, conducted by two independent researchers (R.A. and B.G.), achieving a high agreement level (Kappa = 0.91). Conflicts were resolved in consensus meetings, and a third researcher (A.V.) was consulted when necessary.

-Search Strategy

The search was carried out independently and systematically by two authors (R.A. and B.G.) in the databases MEDLINE/PubMed, EMBASE, Web of Science, Scopus, Cochrane Library and LILACS/VHL, without restrictions on language or publication data. The search was last updated in July 2024. Search terms were combined using Boolean operators to ensure comprehensive coverage of relevant studies ( Table 1). A manual search was also carried out for the same authors in articles published in the following journals from January 2019 to June 2024: International Journal of Oral and Maxillofacial Surgery; Journal of Oral and Maxillofacial Surgery; Journal of Cranio-Maxillofacial Surgery; British Journal of Oral and Maxillofacial Surgery; and oral clinical investigations. In addition, searches were carried out in the gray literature (Clinical trials, open gray, BDTD, Rebec).

-Eligibility Criteria

The study included intervention and observational studies conducted on humans with dental implants or those planning for implants, who had altered lipid levels, including obese patients and those with metabolic syndrome. Studies assessing implant survival, success, loss, peri-implantitis, and measuring serum lipid levels (total cholesterol, LDL, HDL, and triglycerides) were included. Exclusion criteria covered animal studies and those that did not evaluate serum lipid levels or peri-implant outcomes. No restrictions were applied regarding language or publication date.

-Data extraction and synthesis process

Data extracted included author, year, country, study objective, study type, sample, intervention/exposure/case and control groups, pre-operative laboratory and imaging exams, outcomes data such as the number of implant and graft failures, peri-implantitis and the total sample or measures of Relative Risk (RR), Odds Ratio (OR), or correlations reported by the studies. Results of bone loss were presented as averages.

-Risk of Bias and quality assessment

The methodological quality assessment used Joanna Briggs Institute (JBI) tools for quasi-randomized experimental studies, cross-sectional, cohorts, and case-control studies to evaluate study design, conduct, and data analysis. These tools consist of questions to be answered with “yes”, “no”, “unclear” and “no information” (13) The study will be considered low risk if it exhibits low risk across all criteria, high risk when at least one criterion is deemed high risk, and uncertain risk when at least one criterion raises some concerns. A graph was constructed using this instrument, with the assistance of the rob.vis tool.

-Synthesis of Data

Qualitative and quantitative analyses were conducted, including a meta-analysis was conducted to evaluate the peri-implantitis outcome by comparing the likelihood of developing this condition between obese patients with dyslipidemia and non-obese patients with normal lipid levels. The analysis used Review Manager 5.4 software with a random-effects model and the generic inverse variance method. Odds ratios (OR) and their standard errors, calculated from the provided data and confidence intervals, were used. Subgroup analyses were performed based on the type of laboratory test for lipid measurement. The overall estimate included OR with 95% confidence intervals, deemed statistically significant at *p* < 0.05. Heterogeneity was assessed using Qui-square, I², and Tau² statistics.

-Assessment of Evidence Certainty

The Grading of Recommendations Assessment Development and Evaluation (GRADE) (15) approach was used to evaluate the certainty of evidence in this systematic review of the studied outcomes. The assessment was based on five points: risk of bias, inconsistency, indirect evidence, imprecision, and publication bias. In the end, the certainty of evidence was classified into four categories: high, moderate, low, and very low (16).

## Results

The database search strategy resulted in 2714 records, of which 430 were duplicates. Of the 2284 records that were sent for title and summary reading, only 48 were selected for full reading. Of these, eight were included in the systematic review, in addition to three articles from additional searches, totaling 11 studies (Fig. 1).

Of the eleven studies included, six are cross-sectional, one is a retrospective cohort, two are prospective cohorts and two are case-control studies. Six studies are from Europe (three from Italy, one from Spain, one from Belgium and one from Germany), two from Asia (Saudi Arabia), two from South America (Brazil and Peru) and one from Eurasia (Turkey). A total of 1704 patients comprised the sample of interest in this review. Of these, 944 were women and 735 were men. One study did not provide quantitative information about the gender of the population studied (18).  Table 2,  Table 3 presents the characteristics of the included studies, and Table  Table 4 provides the results for each outcome.

Nine of the eleven included studies showed a relationship between serum lipid levels and implant loss, graft loss, bone loss, peri-implantitis, or peri-implant mucositis. Two studies did not find significant results associated with any outcome (5,19). Seven studies used all peri-implant health/disease assessment parameters according to the 2017 World Workshop on the Classification of Periodontal and Peri-implant Diseases and Conditions. Four studies used only the bone loss parameter through the analysis of periapical radiographs (2,5,6,17) and the study by Mayta-Tovalino *et al*. (2019) (2) reported the performance of a periodontal examination without specifying the method.

Peri-Implantitis and Hyperlipidemia

Seven studies analyzed the conditions of peri-implantitis and hyperlipidemia. (1,18-23), among them, patients with and without the condition of metabolic syndrome (20,21), obese and non-obese patients were evaluated (18-20).

The study by Di Murro *et al*. (2019) (20) showed a similar prevalence of peri-implantitis between the groups (OR 1.2014 (95% CI: 0.5798-2.4894) where higher levels of triglycerides and lower HDL cholesterol are present in the case group. This study also considered the confounding factor of smoking and found a higher risk of peri-implantitis in smokers compared to non-smokers. Women were also associated with a higher risk of peri-implantitis.

The study by Papi *et al*. (2018) (21) showed a positive association between metabolic syndrome and peri-implantitis with an OR of 15.25, and peri-implantitis was more prevalent in conditions of higher triglyceride levels and lower HDL cholesterol.

The study by Blanco *et al*. (2021) (23) found a higher chance of peri-implantitis with high total cholesterol and LDL, presenting a significant positive correlation between bone loss and high total cholesterol (r=0.512; *P*<0.001) and LDL (r=0.463; *P*= 0.001), finding no statistical difference about HDL and triglycerides. In this study, smokers were not associated with peri-implantitis.

Ustaoglu, Erdal (2020) (1), evaluated patients with peri-implantitis compared to healthy individuals and found statistically significant triglyceride levels in the case group (case: 148 (107.5); control: 95 (61); < 0.001). No significant differences were found regarding total cholesterol, LDL, and HDL. Clinical assessment parameters (GI, PD, BOP) showed a positive correlation with triglyceride levels.

**Figure 2:**
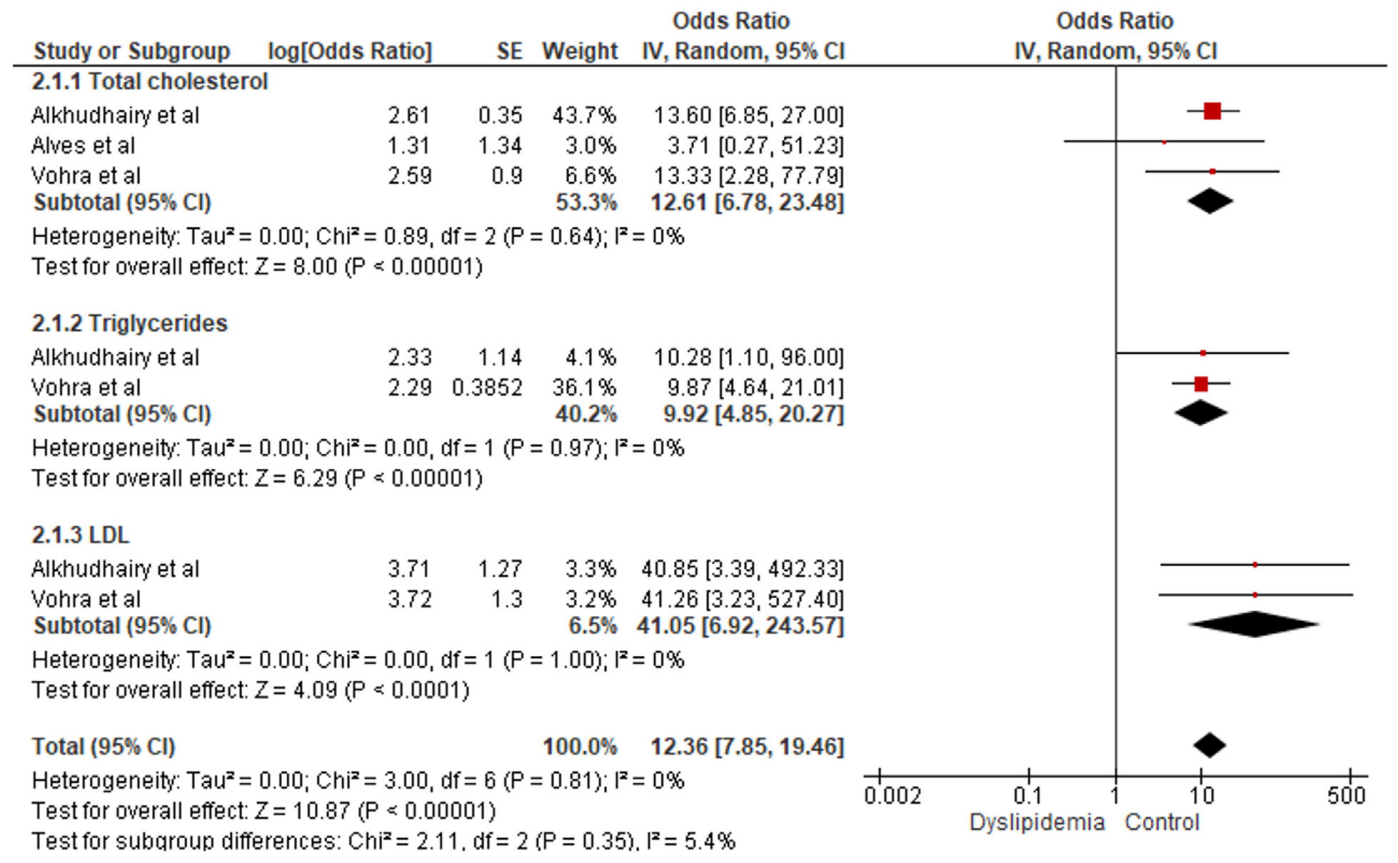
Meta-analysis for peri-implantitis and obesity outcome.

**Figure 3:**
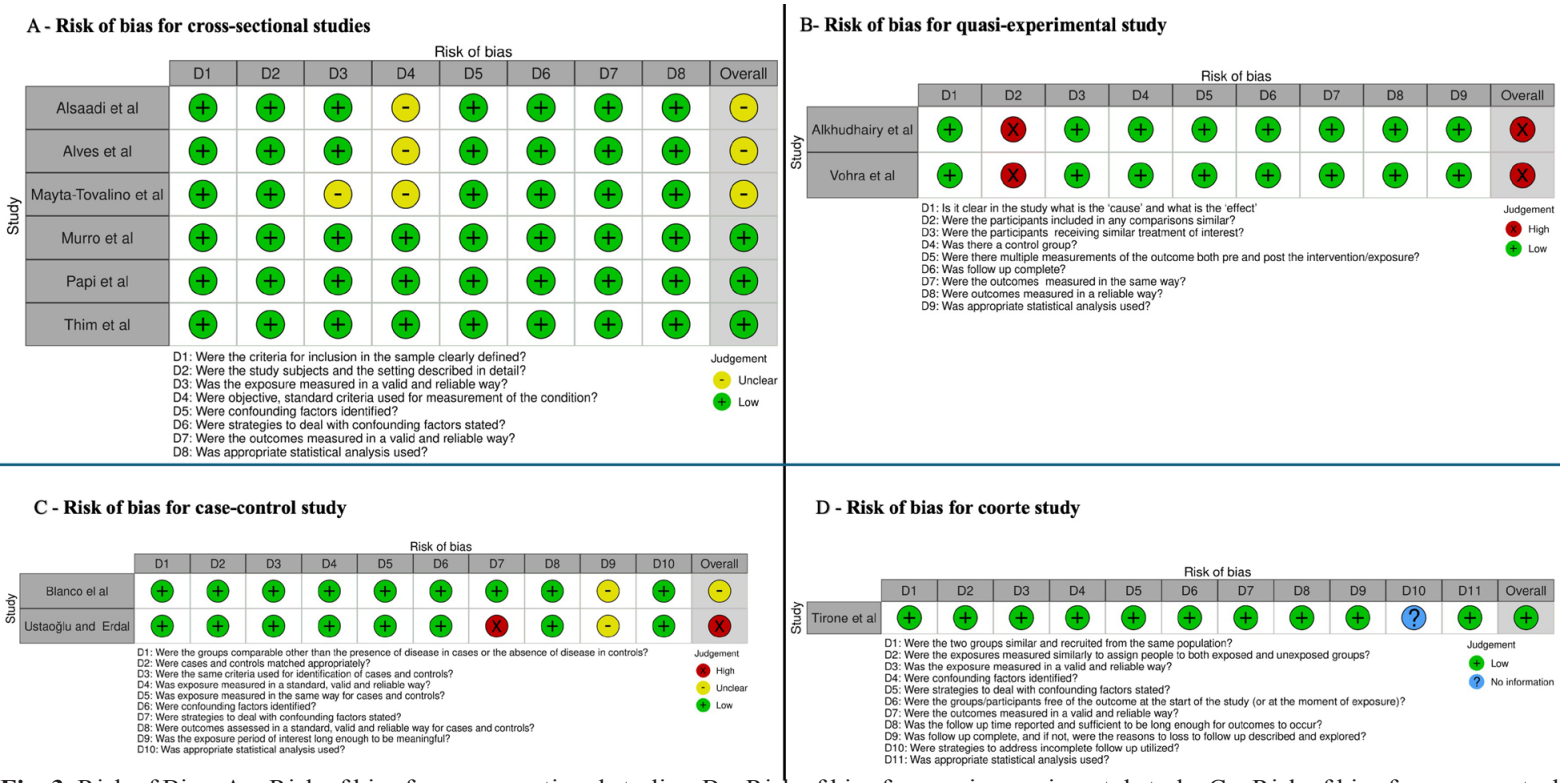
Risk of Bias. A – Risk of bias for cross-sectional studies. B – Risk of bias for quasi-experimental study. C – Risk of bias for case-control study. D – Risk of bias for coorte study.

A meta-analysis was conducted to evaluate the association between peri-implantitis and dyslipidemia and included three studies (18,19,22) (Fig. 2). The results demonstrated a statistically significant odds ratio, indicating a higher likelihood of peri-implantitis in obese patients with elevated lipid levels compared to non-obese patients with normal lipid profiles (OR 12.36; 95% CI: 7.85–19.46; *P* < 0.00001). The findings consistently favored the control group across both the overall estimate and subgroup analyses. No statistical heterogeneity was detected (*p* = 0.81; I² = 0%; Tau² = 0) (Fig. 3).

Loss or survival of implant and hyperlipidemia

Three studies assessed implant failure or survival and its relationship with risk factors. In the study by Alsaadi *et al*. (2008) (5) out of 283 participants, 33 had hypercholesterolemia, and it was not associated with any of the 14 early implant losses. These losses were associated with type 1 diabetes, Crohn’s disease, gastric problems, and total hysterectomy.

Mayta-Tovalino *et al*. (2019) (2) found an implant failure rate of 17.98% over 11 years, and survival was inversely proportional to time. This study identified hypercholesterolemia as a risk factor, with a correlation between high cholesterol and implant failure, along with other factors like implants in bone regeneration areas, osteoporosis, history of periodontitis, bone quality, and prosthetic connection type. This study did not specify the type of cholesterol measured and analyzed. Tirone *et al*. (2016) (17) study did not show a statistically significant association between hypercholesterolemia and implant loss (OR 0.83, *p* = 0.749).

Bone Resorption and Hyperlipidemia

Thim *et al*. (2022) (6) study assessed bone resorption using cone-beam computed tomography in patients planning for implants, comparing patients with hypercholesterolemia to those with optimal, desirable, or borderline cholesterol levels. They observed that bone loss was significantly related to patients with high LDL cholesterol. Smokers in this study represented a confounding factor but were considered in the analyses. These patients showed significantly greater bone loss than non-smokers.

Assessment of Quality and Bias 

The methodological quality assessment revealed that, among the 11 included studies, four had a low risk of bias (06,17,20,21), three had a high risk (1,18,22), and four were classified as having an uncertain risk (02,05,19). The cross-sectional studies had a low to moderate risk (02,05,19), while quasi-experimental studies were considered high-risk due to participant variability. Among the case-control studies, one was classified as high-risk due to the lack of strategies for controlling confounding variables (1), and another had an uncertain risk due to insufficient data on lipid levels (23). The retrospective cohort study was considered low risk of bias (17) (Fig. 3).

## Discussion

The primary research question of this systematic review was: “Is hyperlipidemia a risk factor for peri-implant complications?” Nine out of the eleven included studies demonstrated an association between at least one of the examined outcomes and elevated serum lipid levels. In this context, studies investigating obese individuals with altered lipid profiles compared to those with normal lipid levels were included. Additionally, studies examining patients with metabolic syndrome—a condition characterized by hypertension, visceral obesity, hypertriglyceridemia, low HDL cholesterol, and high fasting glucose—were also considered, comparing them to individuals without the syndrome.

The effect of hyperlipidemia on dental implant osseointegration has not been fully elucidated (24). However, more recently, elevated lipid levels have been associated with changes in bone tissue and periodontal diseases (25,26). Tomofuji *et al*. (2013) (27) report that high levels of oxidative LDL lead to an exacerbated inflammatory response to bacteria, as well as resulting in bone loss. High lipid levels accumulate fat in the bone, and adipocytes release pro-inflammatory substances and osteoclastogenic cytokines (28). These findings reinforce the hypothesis of this systematic review that dyslipidemia appears to be a risk factor for peri-implant complications.

Hyperlipidemia and complications related to implantology, leading to peri-implantitis, implant loss, or graft failure, can also be explained by the interference that high cholesterol levels and their fractions cause in bone metabolism, making it deficient (26). Mandal (2015) (29) in his studies, describes a relationship between dyslipidemia and low bone mineral density, increased osteoclast count, and inhibition of osteoblastic activity.

**Figure 4:**
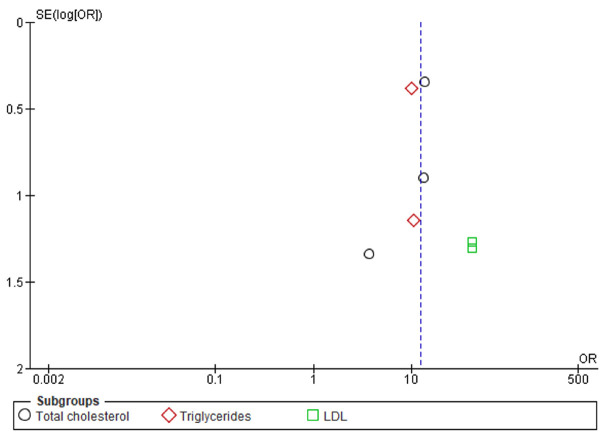
Funnel plot showing potential publication bias in studies on lipid parameters and peri-implant complications.

Regarding peri-implantitis, five out of seven studies addressing this outcome identified a relationship with patients’ lipid profiles. Among these, four reported associations with elevated triglyceride levels (1,18,21,22), three with increased total cholesterol, three with elevated LDL cholesterol (18,22,23) and one with reduced HDL cholesterol (21). One study that found no association (9) identified cholesterol-lowering medication as a confounding factor. The meta-analysis revealed a statistically significant link between dyslipidemia and peri-implantitis. The GRADE assessment indicated a moderate level of certainty in the evidence, demonstrating a strong association (OD > 5) and adequate control of confounding factors in the included studies ( Table 5). However, some imprecision in the results and heterogeneity within the sample were observed. Furthermore, the funnel plot suggests a positive relationship between peri-implantitis and hyperlipidemia (Fig. 4). However, the asymmetry detected in this graph may also indicate the presence of publication bias. Therefore, these results should be interpreted with caution, given the limited number of studies included in the meta-analysis. This limitation may also negatively impact the funnel plot, which is ideally conducted when at least ten studies are included. However, future studies may refine this estimate.

Regarding implant loss and dyslipidemia, only one of the three studies addressing this aspect reported a relationship between the variables. However, the limited number of studies, insufficient data on lipid level measurements, the presence of confounding factors, and the low certainty of evidence according to the GRADE framework preclude definitive conclusions on this outcome. The same applies to bone loss, which, despite presenting a low risk of bias and indicating a tendency toward bone loss associated with high HDL levels, was reported in only a single study with a low certainty of evidence. Further studies are necessary, as they may modify these findings.

Although the studies with patients with metabolic syndrome had a healthy control group (20,21), the presence of the syndrome may have been a confounding factor because it aggregates, in addition to hypercholesterolemia, obesity, and diabetes, which are possible correlated factors to peri-implant disease. Peri-implant mucositis was associated with the syndrome in both studies, while peri-implantitis was associated only in one of them (21). The studies involving obese patients were included because they also presented alterations in lipid levels and compared them with non-obese and healthy patients. However, an association was found between obesity and peri-implantitis (18,19).

 The systematic review by Monteiro *et al*. (2019) (30) warns of a possible association between peri-implant disease and obesity, explained by increased concentrations of inflammatory markers around implants and in the oral cavity of obese patients. In parallel, Gorman *et al*. (31) study shows that obese white adults have more periodontal disease than non-obese individuals, also drawing attention to obesity as a risk factor for periodontal disease. Another systematic review conducted by Nepomuceno *et al*. (32) shows that chronic periodontal disease has been associated with high LDL and triglyceride levels and low HDL levels. These high serum lipid levels seem to be associated with an increased inflammatory response.

 Three studies (5,18,22) showed an association between peri-implantitis or implant loss with high blood glucose levels. However, this was also a confounding factor, as diabetes is a risk factor for periodontal and peri-implant disease. Another confounding factor is the presence of smoking patients, which was found in four included studies (6,20,21,23). Although this condition was excluded in three studies, there is evidence in the literature that smoking presents a significant risk for implant failure (RR: 2.45; CI: 1.42–4.22) in patients who smoke more than 20 cigarettes per day, according to Naseri R, Yaghini J, Feizi (2020) (33).

Regarding the limitations of this systematic review, nine out of the eleven included studies are observational, making them more susceptible to confounding factors in their sampling, design, or execution. The absence of strategies to mitigate these confounding factors, such as accounting for smoking status, contributed to the classification of one study (1) as having a high risk of bias. This assessment was based on the fact that the data correlating outcomes with dyslipidemia did not allow for the determination of whether these patients were smokers—a known risk factor for the studied complications. Another limitation is the small number of studies included in the meta-analysis, primarily due to insufficient data availability. Consequently, the overall sample size was limited, and the wide confidence intervals observed in the forest plot further raise concerns regarding the accuracy and reliability of the results.

This review plays a crucial role in highlighting dyslipidemia as a risk factor potentially associated with peri-implant complications, warranting further attention. A key strength of the presented meta-analysis is its large effect size, which underscores a significant association. However, considering the identified limitations, additional experimental clinical studies are required to compare patients with hypercholesterolemia to those with normal serum lipid levels. This necessitates the measurement of total cholesterol, LDL, HDL, and triglyceride levels while ensuring the standardization of reference values. Moreover, well-defined inclusion and exclusion criteria must be established to minimize confounding variables, which, when present, should always be accounted for.

## Conclusions

This systematic review indicates that dyslipidemia is a statistically significant risk factor for peri-implantitis and may also be a potential risk factor for the other outcomes studied. Although dyslipidemia is not currently considered an absolute contraindication for implant placement, the findings suggest the importance of assessing and managing serum lipid levels in patients undergoing implant rehabilitation or bone regeneration procedures. Preoperative control of dyslipidemia could help reduce the inflammatory potential around implants, promote more efficient bone metabolism, and lower the risk of complications such as peri-implant diseases, implant failure and bone loss.

## Figures and Tables

**Table 1:** Search strategy for each database and journals.

Database	Search strategy
MEDLINE	("Cholesterol"[MeSH Terms] OR "cholesterol, ldl"[MeSH Terms] OR "cholesterol, hdl"[MeSH Terms] OR "Hypercholesterolemia"[MeSH Terms] OR ("Hypercholesterolemia"[All Fields] OR "Cholesterol"[All Fields] OR "high cholesterol"[All Fields] OR ("dislipidemia"[MeSH Terms] OR "dislipidemias"[All Fields]))) AND ("Dental Implants"[MeSH Terms] OR "Alveolar Bone Grafting"[MeSH Terms] OR "Autografts"[MeSH Terms] OR "Heterografts"[MeSH Terms])
EMBASE	('cholesterol'/exp OR '3 hydroxy 5 cholestene' OR '3beta hydroxy 5 cholestene' OR '3beta hydroxycholest 5 ene' OR '5 cholesten 3beta ol' OR 'beta cholesterol' OR 'cholest 5 en 3beta ol' OR 'cholest 5 ene 3 ol' OR 'cholesterin' OR 'cholesterine' OR 'cholesterol' OR 'cholesterol release' OR 'dythol' OR 'nsc 8798' OR 'hypercholesterolemia'/exp OR 'cholesteremia' OR 'cholesterinemia' OR 'cholesterolemia' OR 'hypercholesteremia' OR 'hypercholesterinaemia' OR 'hypercholesterinemia' OR 'hypercholesterolaemia' OR 'hypercholesterolemia' OR 'cholesterol diet'/exp OR 'cholesterol diet' OR 'high cholesterol diet') AND ('tooth implant'/exp OR 'bicon' OR 'grafton' OR 'straumann mini' OR 'straumann pure' OR 'swish active' OR 'swish tapered' OR 'variobase' OR 'dental implant' OR 'dental implants' OR 'implant, teeth' OR 'implant, tooth' OR 'implants, teeth' OR 'implants, tooth' OR 'intramucosal dental implant' OR 'teeth implant' OR 'teeth implants' OR 'tooth implant' OR 'tooth implants' OR 'bone graft'/exp OR 'autograft'/exp OR 'xenograft'/exp OR 'alveolar bone grafting'/exp)
WEB OF SCIENCE	(((TS=(Cholesterol)) OR TS=(Hypercholesterolemia)) OR TS=("high cholesterol"[)) OR TS=(dislipidemia) AND ((((((TS=("Dental Implants")) OR TS=("tooth implant")) OR TS=("bone grafting")) OR TS=(Heterografts)) OR TS=(xenografts)) OR TS=("alveolar bone grafting"))
COCHRANE	Cholesterol OR Hypercholesterolemia OR "high cholesterol" OR dislipidemia in Title Abstract Keyword AND "Dental Implants" OR "tooth implant" OR "bone grafting" OR Heterografts OR xenografts OR "alveolar bone grafting" in Title Abstract Keyword
SCOPUS	TITLE-ABS-KEY ( cholesterol OR hypercholesterolemia OR "high cholesterol" OR dislipidemia ) AND TITLE-ABS-KEY ( "Dental Implants" OR "tooth implant" OR "bone grafting" OR autograft OR heterografts OR xenografts OR "alveolar bone grafting") Edit Save Set alert
Journals	International Journal of Oral and Maxillofacial Surgery; Journal of Oral and Maxillofacial Surgery; Journal of Cranio-Maxillofacial Surgery; British Journal of Oral and Maxillofacial Surgery; and Clinical Oral Investigations

**Table 2:** Presents the characteristics of the included studies.

Study/Year/Country	Study Type	Objective	Sample	Sex/Age	Surgical Procedure	Lipid Parameters	Adjustment for Confounding Factors	Conclusion
Alkhudhairy et al., 2018 Arábia Saudita	Quasi-experimental study	Comparing peri-implant parameters between obese and non-obese individuals.	38 patients	16 female; 22 male	Dental Implants	Total cholesterol, LDL, HDL, and triglycerides	YES	Marginal bone loss was associated with high lipid levels of total cholesterol, LDL, and triglycerides.
Alsaadi et al., 2008Bélgica	Cross-sectional study	Identifying systemic, local, and intraoral factors related to the incidence of early implant failure.	283 patients; 720 implants (total)	187 female; 96 male, average age of 56.2 years (18–86)	Dental implants >=7mm installed in two stages	Cholesterol, unspecified	YES	High cholesterol was not associated with a higher risk of implant loss.
Alves et al., 2020Brasil	Cross-sectional study	Identifying systemic risk indicators associated with peri-implant mucositis and peri-implantitis.	1 patients 360 implants	50 female; 21 male <56 years - 46.6%	Dental implants with a minimum of 6 months in function	Cholesterol, unspecified	YES	Peri-implantitis and peri-implant mucositis were not related to high cholesterol. Obesity and hypertension were associated with peri-implantitis but not with mucositis.
Blanco el al., 2021Espanha	Case-control study	Comparing the inflammatory and lipid profiles of patients with peri-implantitis.	47 patients 20 implants	24 female; 23 male, Mean age 57.4 years	Dental implants without specifying time in function	Total cholesterol, LDL, HDL, and triglycerides	YES	High total cholesterol and LDL were correlated with a higher risk of peri-implantitis.
Mayta-Tovalino et al., 2019Peru	Cross-sectional study	Analyzing risk factors and survival of osseointegrated implants in patients from public and private services.	431 patients 1279 Implants	218 female; 213 male	Dental implants without specifying time in function	Cholesterol, unspecified	YES	High cholesterol was identified as a risk factor for implant success/survival.
Di Murro et al., 2019Itália	Case-control study	Assessing the association between peri-implantitis and metabolic syndrome.	41 patients 132 implants	23 female; 18 male; mean age of 62±13 years	Dental implants with a minimum of 5 years in function	HDL and triglycerides	YES	Metabolic syndrome was not associated with peri-implantitis but was associated with mucositis. Patients with metabolic syndrome had lower levels of HDL, higher levels of triglycerides, and a larger abdominal circumference.
Papi et al., 2019Itália	Cross-sectional study	Detecting the frequency and severity of peri-implantitis in patients with metabolic syndrome.	183 patients 567 implants	112 female; 71 male, 66.08 ± 10.42 years	Dental implants with a minimum of 5 years in function	HDL and triglycerides	YES	Metabolic syndrome showed a positive correlation with peri-implantitis.

*Probing Depth (PD); Bleeding on Probing (BOP); Plaque Index (PI); Marginal Bone Loss (MBL)

**Table 3:** Presents the characteristics of the included studies.

Study/Year/Country	Study Type	Objective	Sample	Sex/Age	Surgical Procedure	Lipid Parameters	Adjustment for Confounding Factors	Conclusion
Thim et al., 2022Alemanha	Cross-sectional study	Investigating the association between bone resorption in areas to receive implants and serum levels of LDL cholesterol and vitamin D.	163 patients	100 females 63 males Mean age 53 years	Planning for implants	LDL	YES	The study demonstrated an association between bone loss and high levels of LDL cholesterol.
Tirone et al., 2016	cohort study	To assess the impact of hypercholesterolemia on dental implant and bone graft failure by analyzing the association between elevated total cholesterol (TC) levels and postoperative complications.	268 patients	121 females 106 males Mean age 56,9 years	Dental implant surgery and bone augmentation, including sinus lift, guided bone regeneration, and block grafting.	Total cholesterol (TC)	YES	This study found a link between high cholesterol levels and graft failures but not implant failures.
Ustaoglu, Erdal, 2020Turquia	Cross-sectional study	Evaluate the risk factors for peri-implantitis.	156 patients	93 female; 61 male	Dental implants with a minimum of 3 years in function	Total cholesterol, LDL, HDL, and triglycerides	NO	There was a correlation between triglyceride levels and peri-implantitis. However, there was no correlation between total cholesterol, HDL, LDL, and peri-implantitis.
Vohra et al., 2017 Arábia Saudita	Quasi-experimental study	Evaluate whether peri-implant diseases in individuals with varying degrees of obesity are associated with changes in lipid levels.	84 patients	Obese class I: 49.3 years (42–56); Obese class II: 51.8 years (40–54); Obese class III: 50.4 years (45–59); non-obese: 52.1 years (39–58)	Dental implants with a minimum of 3 years in function	Total cholesterol, LDL, HDL, and triglycerides	YES	Peri-implantitis was associated with high total cholesterol, LDL, and triglycerides. There was no association with HDL.

*Probing Depth (PD); Bleeding on Probing (BOP); Plaque Index (PI); Marginal Bone Loss (MBL)

**Table 4:** Provides the results for each study outcome.

Study/Year/Country	Study Type	Sample	Exposure/Case	Control	Implant Loss	Bone Loss	Peri-Implantitis
Alkhudhairy et al., 2018 Arábia Saudita	Na-experimental study	38	20 Obese	18 Non-obese	Not Aplicate	Not Applicable	Marginal ign loss was associated with elevated levels of total cholesterol (OR=2.61; 1.21, 4.77; p=0.031), LDL (OR=3.71; 1.12, 6.12; P=0.077), and Triglycerides (OR=2.33; 1.56, 6.02; P=0.014).
Alsaadi et al., 2008Bélgica	Cross-sectional study	283	Not Applicable	Not Applicable	33 participants had high cholesterol, and none of these patients experienced implant loss. There were 14 early implant losses out of a total of 720 implants (1.9%).	Not Applicable	Not Applicable
Alves et al., 2020Brasil	Cross-sectional study	71	16 Obese (22.5%) 15 High Cholesterol (21.1%)	55 Non-obese with normal cholesterol	Not Aplicate	Not Applicable	The prevalence of peri-implantitis was 16.9% (12 patients). Peri-implantitis was not associated with hypercholesterolemia (OR 1.31 (0.31–5.58), p=0.50). Peri-implantitis was associated with obesity (OR 3.12 (0.83–11.69) P=0.09).
Blanco et al., 2021Espanha	Case-control study	47	16 patients with peri-implantitis	31 Healthy patients	Not Aplicate	Not Aplicate	Total cholesterol and LDL were significantly higher in the case group. Positive correlation between ign loss and total cholesterol (r=0.512; P<0.001). Positive correlation between ign loss and LDL (r=0.463; P=0.001).
Mayta-Tovalino et al., 2019Peru	Cross-sectional study	411	Not Applicable	Not Applicable	42 patients had high cholesterol. There were 23 implant losses. There was a correlation between high cholesterol and implant failure: OR 5.1 (1.02–25.44) P=0.046	Not Applicable	Not Applicable
Di Murro et al., 2019Itália	Case-control study	41	22 Metabolic Syndrome	19 Healthy	Not Applicable	Not Applicable	Prevalence of peri-implantitis was 35.2% (25/71 implants) in patients with metabolic syndrome. In the control group, this prevalence was 31.1% (19/61 implants) with no statistical difference OR = 1.2014 (0.5798-2.4894).
Papi et al., 2019Itália	Cross-sectional study	183	84 patients (45.9%) with Metabolic Syndrome	99 patients (54.1%) healthy	Not Applicable	Not Applicable	36.5% (31/84) of patients with metabolic syndrome had peri-implantitis. In the control group, 26.2% (26/99) had peri-implantitis. There was a higher chance of peri-implantitis in the exposed group with na OR of 15.26 (2.90-80.20; p<0.005).
Tirone et al., 2016Itália	Retrospective cohort study	227	39 patients with high cholesterol (>200)	88 patients with cholesterol < 200	There were 20 implant losses in 14 patients. Among them, 8 had high cholesterol (>200), with no statistically significant relationship (OR 0.83, p=0.749).	Not Applicable	Not Applicable
Thim et al., 2022	Cross-sectional study	163 patients	LDL levels	YES	Not Applicable	Yes, associated with high LDL levels	Not Applicable
Ustaoglu, Erdal 2020Turquia	Cross-sectional study	156	58 patients with peri-implantitis 49 patients with peri-implant mucositis	49 Healthy	Not Applicable	Not Applicable	Serum levels of total cholesterol, LDL, and HDL showed no statistical difference when comparing the group with peri-implantitis and the groups with mucositis and healthy individuals. Triglyceride serum levels were higher, with statistical significance in the peri-implantitis group compared to the others. Vitamin D values were significantly higher in healthy patients. Patients with peri-implantitis had deficiency values of this vitamin.
Vohra et al., 2017 Arábia Saudita	Quasi-experimental study	84	25 Class-I Obese (BMI 27.5–34.9 kg/m2) 39 implants 22 Class-II Obese (BMI 35–39.9 kg/m2) 35 implants 12 Class-III Obese (BMI >40 kg/m2) 26 implants	25 Non-obese (BMI 18.5–22.9 kg/m2)	Not Applicable	Not Applicable	Peri-implantitis was associated with high total cholesterol (OR=2.59; 1.18,4.72; P=0.032), LDL (OR=3.72; 1.01, 6.11; P=0.075), and triglycerides (OR=2.29; 1.52, 6.88; P=0.017). There was no association with HDL.

*Probing Depth (PD); Bleeding on Probing (BOP); Plaque Index (PI); Marginal Bone Loss (MBL)

**Table 5:** Assessment of certainty of evidence. Question: Are dyslipidemia are a risk factor off peri-implant complications?

Certainty assessment								Certainty
Outcome	№ of studies	Study design	Risk of bias	Inconsistency	Indirect evidence	Inaccuracy	Other considerations	
Peri-implantitis	06	observational study	Serious	Serious	Don't record	Serious	Strong association: potential confounders reduce effect; dose-response gradient observed	ѳѳѳοModerate
Loss or survival	03	observational study	Don't record	Serious	Don't record	Serious	All potential confounding factors would reduce the demonstrated effect	ѳοοο Very low
Bone resorption	01	observational study	Don't record	Serious	Don't record	Serious	All potential confounding factors would reduce the demonstrated effect	ѳοοο Very low

CI: Confidence interval

## Data Availability

The datasets used and/or analyzed during the current study are available from the corresponding author.
